# Effects of the Glass-Forming Ability and Annealing Conditions on Cocrystallization Behaviors via Rapid Solvent Removal: A Case Study of Voriconazole

**DOI:** 10.3390/pharmaceutics12121209

**Published:** 2020-12-14

**Authors:** Si Nga Wong, Susan Wing Sze Chan, Xuexin Peng, Bianfei Xuan, Hok Wai Lee, Henry H. Y. Tong, Shing Fung Chow

**Affiliations:** 1 Li Ka Shing Faculty of Medicine, Department of Pharmacology and Pharmacy, The University of Hong Kong, Pokfulam, Hong Kong, China; snwongab@connect.hku.hk (S.N.W.); cwc0131@connect.hku.hk (S.W.S.C.); bxuan@connect.hku.hk (B.X.); rhwlee@hku.hk (H.W.L.); 2 School of Pharmacy, University College London, London WC1N 1AX, UK; lakepengxuexin@gmail.com; 3 School of Health Sciences and Sports, Macao Polytechnic Institute, Macao, China; henrytong@ipm.edu.mo

**Keywords:** cocrystal, amorphous, voriconazole, glass-forming ability, annealing temperature, rotary evaporation

## Abstract

The kinetic entrapment of molecules in an amorphous phase is a common obstacle to cocrystal screening using rapid solvent removal, especially for drugs with a moderate or high glass-forming ability (GFA). The aim of this study was to elucidate the effects of the coformer’s GFA and annealing conditions on the nature of amorphous phase transformation to the cocrystal counterpart. Attempts were made to cocrystallize voriconazole (VRC) with four structural analogues, namely fumaric acid (FUM), tartaric acid (TAR), malic acid (MAL), and maleic acid (MAE). The overall GFA of VRC binary systems increased with decreasing glass transition temperatures (T_g_s) of these diacids, which appeared as a critical parameter for predicting the cocrystallization propensity such that a high-T_g_ coformer is more desirable. A new 1:1 VRC-TAR cocrystal was successfully produced via a supercooled-mediated re-cocrystallization process, and characterized by PXRD, DSC, and FTIR. The cocrystal purity against the annealing temperature displayed a bell-shaped curve, with a threshold at 40 °C. The isothermal phase purity improved with annealing and adhered to the Kolmogorov–Johnson–Mehl–Avrami kinetics. The superior dissolution behavior of the VRC-TAR cocrystal could minimize VRC precipitation upon gastric emptying. This study offers a simple but useful guide for efficient cocrystal screening based on the T_g_ of structurally similar coformers, annealing temperature, and time.

## 1. Introduction

The application of rapid solvent removal via rotary evaporation has gained increasing popularity for screening kinetically stable pharmaceutical cocrystals, which cannot be obtained by neat grinding, slow evaporation, slurry conversion, etc. It is deemed an efficient and easy-to-use approach for circumventing the inherent cocrystal metastability through creating a sufficiently high degree of solute supersaturation in solution [1,2]. Nevertheless, at the same time, it is not uncommon to encounter amorphization during rotary evaporation [3,4,5] since the rapid elimination of solvent can kinetically entrap the drug and coformer in amorphous content and prevent these solute molecules from rearranging into a long-range order structure of crystals. Such an effect is more pronounced for metastable systems, where the phase purity of cocrystals correlates to the solvent evaporation rate [6]. Although amorphization represents an attractive formulation strategy for improving the apparent solubility and dissolution behavior of active pharmaceutical ingredients (APIs), amorphous solids often possess a relatively low physical stability and are prone to phase transformation to their crystalline counterparts during storage, as depicted by Ostwald’s rule of successive stages [7,8]. In recent years, different techniques have emerged in order to enhance the stability of amorphous drugs, such as the preparation of polymer-based glass solutions, as well as the use of mesoporous silicon and silica-based carriers [9,10,11]. The major drawbacks of these approaches are issues associated with a limited drug loading capacity [12]. Coamorphous systems, consisting of an API and one or more low molecular weight coformers, represent an attractive alternative for achieving solubility enhancement through intermolecular interactions [9,13]. Despite no conclusion having been drawn about whether cocrystal formation is favored over coamorphous systems at present [9], it should be noted that a cocrystal retains its distinct value in oral dosage form production and storage in light of its capability to simultaneously improve not only the dissolution rate, but also the tableting behavior and hygroscopicity, etc. [14].

Given that the processing temperature in rotary evaporation can be higher than the glass transition temperature (T_g_) of the amorphous binary mixture of API and coformer, especially when those with low T_g_s are employed, the resulting product has a great propensity to exist in the supercooled rubbery state. The gel-like nature of supercooled liquids renders them poorly processed and characterized. More importantly, such a phenomenon might be regarded as a sign of failure towards cocrystallization due to the unpredictable devitrification at ambient conditions, which may eventually occur in months or years [15]. The challenge of controlling the crystallization behavior in glass-forming liquids is associated with the intrinsic glass-forming ability of the compound. The glass-forming ability (GFA) describes the ease of amorphization of compounds and is generally divided into three classes, based on their crystallization tendency from the supercooled melt during a differential scanning calorimetry (DSC) heat-cool-heat cycle [16,17]. Class I compounds, i.e., non-glass formers, readily crystallize from the supercooled melt upon cooling at a temperature lower than the melting point. In contrast, Class II and Class III compounds both form amorphous materials upon cooling the melt, while only Class III compounds exhibit no sign of recrystallization on heating the melt-quenched materials, especially those with high molecular weights and complex structures.

Compared with the glassy state counterpart, supercooled liquid is less viscous with a higher molecular mobility, leading to faster recrystallization in a single-component system. However, in amorphous binary mixtures, the recrystallization rate of the API from supercooled liquid can be manipulated by the inclusion of coformer, which has been exploited as a potential method for improving the physical stability of the neat amorphous drug instead of using polymers in amorphous solid dispersion [18,19,20]. It is worth noting that previous research studies have mainly reported the recrystallization of coamorphous systems to individual constituents after storage [21]. Nonetheless, the annealing conditions (e.g., annealing temperature and storage period) for cocrystal screening and formation have not been subjected to in-depth investigations. To this end, this study aims to provide deeper understandings regarding the phase transitions between supercooled amorphous and cocrystal states. As a prerequisite to developing a robust re-cocrystallization process to mitigate the unwanted amorphization caused by rotary evaporation, this study will investigate the (i) effect of coformer on modulating the overall GFA in the amorphous binary mixture, and (ii) effect of the annealing temperature and annealing time on the recrystallization efficiency and cocrystal purity. Properly understanding the roles of the abovementioned parameters may offer a clue on how to perform a more effective and successful cocrystal screening of amorphous binary or coamorphous systems exhibiting strong GFA.

**Figure 1:**
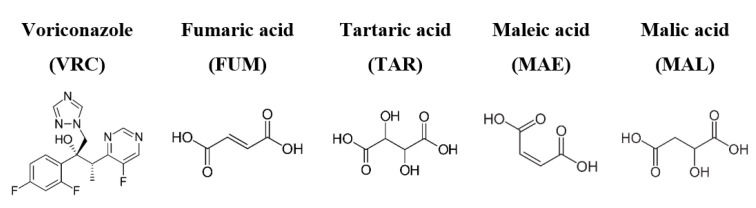
Chemical structures of voriconazole (VRC) and the chosen C4 diacid coformers.

In this study, voriconazole (VRC) ((2R,3S)-2-(2,4-difluorophenyl)-3-(5-fluoropyrimidin-4-yl)-1-(1H-1,2,4-triazol-1-yl)butan-2-ol) was chosen as the model compound. It is used as the frontline therapy against common fungal pathogens, ranging from *Aspergillus*, *Candida*, and *Scedosporium* to *Fusarium* spp. [22,23,24]. Despite its broad-spectrum activity, the efficacy of oral VRC formulations via systemic administration is hampered by the subtherapeutic concentration owing to its poor aqueous solubility (<0.1 mg/mL) [25]. Although cocrystal engineering shows promise for modifying the physicochemical properties of problematic drugs, relevant successful cases of VRC are currently limited. From industrial experience, VRC is considered a highly plastic and elastic material which is difficult to process [26], indicating the propensity of being amorphous during manufacturing. Amorphous VRC has a low T_g_, i.e., 11.9 °C, and was previously shown to behave as strong supercooled liquid with moderate GFA [27]. To investigate the cocrystallization potential of VRC via rotary evaporation in relation to its GFA in binary mixtures under different annealing conditions, a series of four-carbon dicarboxylic acids (C4 diacids), namely fumaric acid (FUM), tartaric acid (TAR), malic acid (MAL), and maleic acid (MAE), with distinct T_g_s were employed as the coformers (Figure 1). All of them are pharmaceutically approved excipients, while TAR also exerts certain inhibitory effects on the fungal growth of *Aspergillus flavus* and *Penicillium purpurogenum*, etc. [28]. Given the structurally similar nature of the coformers, we believe that the findings may offer important insight into the interplay between GFA and annealing conditions, and can open up a new direction for the successful screening of elusive cocrystals and advance the manufacturing efficiency by means of rotary evaporation.

## 2. Materials and Methods

### 2.1. Materials

Voriconazole (VRC, >99.5%) was purchased from Yick Vic Chemicals & Pharmaceuticals Limited, Hong Kong, China. l-(+)-Tartaric acid (TAR), fumaric acid (FUM, ≥99%), maleic acid (MAE, ≥99%), and L-(-)-Malic acid (MAL, ≥99%) were supplied by Sigma-Aldrich (St. Louis, MO, USA). Ethanol of an analytical grade was sourced from VWR BDH Chemicals (VWR International S.A.S., Fontenay-sous-Bois, France). Potassium bromide (KBr) for FTIR analysis was obtained from J&K Scientific Limited, Beijing, China. Water was purified through the Barnstead Ultrapure Purification System (Thermo Fisher Scientific, Waltham, MA, USA).

### 2.2. Preparation of Cocrystals

A total of 300 mg of 1:1 equimolar VRC and C4 diacid (FUM: 0.64 mol, TAR: 0.6 mol, MAE: 0.64 mol, and MAL: 0.62 mol) was dissolved in 100 mL ethanol, followed by sonication, until a clear solution was obtained. Ethanol was then removed using an EYELA N-1300 rotary evaporator (EYELA Corporation Ltd., Shanghai, China) in a vacuum environment of −0.1 MPa, achieved by the EYELA A-1000S Aspirator Pump (EYELA Corporation Ltd., Shanghai, China), with a rotating speed of 20 rpm and a water bath temperature of 60 °C, resulting in an average solvent evaporation rate of around 0.19 mL/s. After solvent dryness, the round bottom flasks were sealed with aluminum foil to minimize the effect of moisture, and dried at different annealing temperatures (4, 20, 40, 60, and 80 °C). Subsequently, the products were collected in parafilm-wrapped plastic vials at designated time points for further analysis in triplicate.

### 2.3. Differential Scanning Calorimetry (DSC)

Thermal analysis was conducted by a TA DSC 250 differential scanning calorimeter (TA Instruments, New Castle, DE, USA) with nitrogen as purge gas at 50 mL/min. Pure indium was used for routine calibration of the enthalpy and cell constant. Samples in the range of 1 to 3 mg were accurately weighed and encased in Tzero Aluminum Hermetic pans (TA Instruments, New Castle, DE, USA) with a pinhole-vented lid if required and heated at a scanning rate of 10°C/min to generate the thermogram. The TA Trios Software (v5.1.1, TA Instruments, New Castle, DE, USA) was used for data analysis. The glass formation of VRC-TAR was investigated by a DSC heat-cool-heat cycle using its physical mixture: Heating to a temperature above the peak melting point at a ramp rate of 10 °C/min, followed by quench cooling to −70 °C at a ramp rate of 50 °C/min. Detection of the T_g_ was then achieved by heating the system again at a heating rate of 10 °C/min.

### 2.4. Powder X-ray Diffraction (PXRD)

The X-ray powder diffraction data were collected using a Panalytical X-ray diffractometer (Philips X’Pert PRO, Eindhoven, The Netherlands), equipped with Cu−Kα radiation (λ = 1.5406 Å, 40 kV, 40 mA). The sample was evenly packed in a custom-made aluminum holder with a 2 mm depth and scanned with a 2θ interval from 2 to 40° at a 0.04° step size with a 4° per minute scanning speed. The amplitude (A) and full width at half maximum (FWHM) of the XRD characteristic peaks were obtained by Gaussian fit using OriginPro software (2020, OriginLab Corporation, Northampton, MA, USA), in order to calculate the peak area.

### 2.5. Fourier-Transform Infrared Spectroscopy (FTIR)

An FTIR spectrometer (ALPHA, Bruker, Ettlingen, Germany) in diffuse reflectance mode was used to obtain FTIR spectra. A small quantity of sample was gently ground with IR grade potassium bromide (KBr) at a ~1:100 *w*/*w* ratio using a marble pestle and mortar. The sample was subsequently compressed into a thin translucent disc under two tons of force using a Mini-Pellet Press (Specac Limited, Orpington, UK). A total of 16 scans were performed in the range of 4000 to 500 cm^−1^ at a resolution of 4 cm^−1^ for each sample. The data generated were analyzed by the built-in software.

### 2.6. High Performance Liquid Chromatography (HPLC)

The concentrations of VRC and TAR in the solubility and dissolution study were examined using an HPLC system equipped with a diode array detector (Agilent 1200 series, Agilent Technologies, Wilmington, DE, USA) and an Agilent Zorbax Eclipse Plus C18 column (5 μm, 250 mm × 4.6 mm, Agilent Technologies, Wilmington, DE, USA) in isocratic conditions. For VRC, the mobile phase consisted of a mixture of acetonitrile and 0.5% formic acid solution (50:50 *v*/*v*), as reported by Liao et al. [29]. For TAR, a mobile phase made up of 50 mM phosphate buffer (pH = 2.2 adjusted with phosphoric acid) was used. The detection wavelengths were 256 and 214 nm with retention times of 5.6 and 2.9 min for VRC and TAR, respectively. A 25 μL aliquot of each sample solution was injected and ran at an isocratic flow rate of 1 mL/min at room temperature.

### 2.7. Powder Dissolution Study

The dissolution study was carried out in triplicate using the Copley Dissolution Tester DIS8000 (Copley Scientific Limited, Nottingham, UK). Considering that a 50 mg VRC tablet is often recommended for critically ill patients with fungal infections, the release profiles of 50 mg of VRC powder and 71.5 mg of VRC-TAR cocrystal powder (equivalent to a 50 mg VRC content) prepared by rotary evaporation were examined. The variation of the particle size and morphology of the samples was minimized using a standard testing sieve with a diameter of 63 μm (VWR International, West Chester, NY, USA). The morphologies of the sifted samples were observed under a phase contrast microscope (Nikon ECLIPSE, TS100, Tokyo, Japan). The powders were poured into dissolution vessels containing 900 mL of 0.1N HCl (pH 1.2) and phosphate buffer (pH 6.8) solution, respectively, with a paddle rotation speed of 50 rpm at 37 °C. A total of 5 mL of the dissolution medium was withdrawn at specific time points, i.e., 2.5, 5, 10, 15, 20, 30, 45, 60, 90, and 120 min, and replaced with an equal volume of fresh buffer medium. The sample solution was filtered through a nylon syringe filter with a pore size of 0.45 μm and subjected to HPLC analysis, as described in Section 2.6.

## 3. Results and Discussion

### 3.1. Role of C4 Diacid Coformers in Altering the Glass-Forming Ability and Crystallization Tendency of VRC

**Figure 2:**
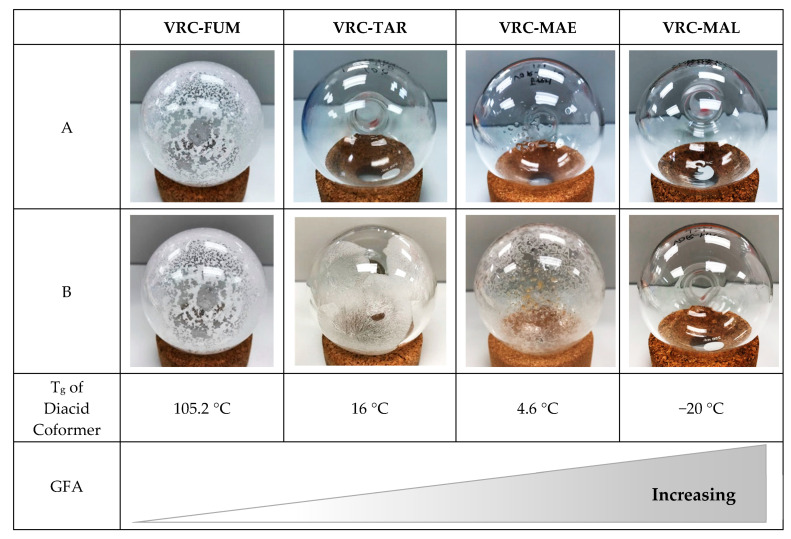
The physical appearance of the products presented in the round bottom flask right after the completion of evaporation (**A**) and 3 days after aging at 60 °C (**B**).

It was previously reported that VRC could cocrystallize with FUM via liquid-assisted grinding with methanol, which modulated the mechanical property of VRC [30]. Surprisingly, the potential of VRC cocrystal formation with TAR, MAE, and MAL has not received attention, despite the fact that they share highly similar chemical structures. Here, we aim to fill this gap by means of rotary evaporation and relate the cocrystallization outcome to the glass-forming ability of resultant products. A new 1:1 VRC-TAR cocrystal was successfully derived in the present study (see Section 3.3). To gain a better understanding on the crystallization behavior of the products obtained, their visual appearance was captured right after the completion of rotary evaporation (Figure 2A). When subjected to a processing temperature of the water bath of 60 °C, 1:1 VRC-FUM readily crystallized within a few minutes. However, VRC-TAR, VRC-MAE, and VRC-MAL in 1:1 stoichiometry appeared as either optically transparent amorphous material or a mixed phase with a microcrystalline dispersion in an amorphous mass, of which their solid state properties were not able to be timely characterized due to the high instability and stickiness nature of the product prior to annealing. After annealing at a temperature of 60 °C for 3 days (Figure 2B), only the VRC-TAR system underwent an obvious phase transformation from an amorphous mixture to a crystalline phase, while VRC with MAE and MAL remained as supercooled liquid.

In the context of the glass-forming ability (GFA), the molecular weight is an important descriptor, as larger compounds are generally less prone to crystallization [31]. With a relatively high molecular weight (i.e., 349.31 g/mol) among pharmaceutical compounds, VRC has previously exhibited a moderate GFA [16,27]. The finding herein demonstrated that the GFA of VRC could be tuned by rapid solvent removal with the selected coformers. TAR, MAE, and MAL enhanced the GFA of VRC to different extents, such that it was easier to convert VRC into amorphous than pure VRC (Figure 2). Moreover, the cocrystallization tendency of these systems is apparently associated with the glass transition temperature (T_g_) of the coformers, which is a common kinetic parameter employed for predicting the temperature dependence of the molecular mobility and physical stability of amorphous pharmaceutical solids during formation and storage [32,33]. The T_g_s of the diacids are listed in Table 1 [34]. Due to the high resistance of FUM and MAE to being amorphized when melt-quenched, their theoretical T_g_ values were estimated using an empirical model where T_g_ ≈ (2/3)T_m_ (T_g_: glass transition temperature and T_m_: melting temperature, in degree Kelvin) [35,36]. The T_g_ derived from this relationship has been shown to be in good agreement with the experimental values for a wide variety of organic compounds [37,38,39]. As depicted in Figure 2, it can be seen that the overall GFA of VRC binary systems increased with the decreasing T_g_ of the diacid coformer. VRC with FUM, which has the highest T_g_ (105.2 °C) among the four diacids, showed non-glassy Class I GFA behavior and readily cocrystallized from the supercooled melt, regardless of the effect of annealing. In contrast, attempts to cocrystallize VRC with TAR, which has the second highest T_g_ (16 °C), rendered VRC that exhibited Class II GFA behavior via rotary evaporation. This is inferred based on the common trend that the melt-quenched amorphous phase of Class II GFA compounds could crystallize when being stored above the T_g_, as opposed to Glass III compounds. Without an obvious sign of recrystallization, the diacids with relatively low T_g_s, i.e., MAE (4.6 °C) and MAL (−20 °C), on the other hand, resulted in Class III GFA behavior [16,40]. It has been shown that for co-milling and spray-drying, which are also major sources of inducing amorphization, high T_g_ excipients confer functional advantages for stabilizing the amorphous drugs [41,42,43,44,45]. However, the opposite trend was observed in our study, i.e., the crystallization tendency was positively correlated with the T_g_ of the coformer (Figure 2). Although MAL exhibited the lowest T_g_ among the selected C4 diacids, VRC-MAL was exceptionally stable with high GFA and retained in transparent amorphous mass after 14 days of aging. Consequently, to control an unwanted amorphization of APIs triggered by rotary evaporation, especially those with moderate or high GFA, a structurally similar coformer with a higher T_g_ might be more desirable. This discrepancy is perhaps not surprising and can be attributed to the different types of intermolecular interaction involved. Polymeric carriers, which are usually employed in fabricating amorphous solid dispersions, generate less specific molecular interactions, while the molecular recognition is stronger and more directional to influence the crystallization tendency when stoichiometric hydrogen bonds are formed in cocrystal.

The advantage of selecting high-T_g_ coformers in cocrystal screening might have an important implication for the elusiveness of some cocrystal systems, for example, aliphatic dicarboxylic acids with variable carbon chain lengths (i.e., HOOC–(CH_2_)_n_–COOH) are regarded as the most commonly used coformers. Historically, many research groups have reported unsuccessful attempts to cocrystallize long-chain acids (*n* = 7–10) with different pharmaceutical compounds, such as itraconazole and ketoconazole, etc., despite their structural resemblance to other short-chain acids [47,48,49,50,51,52,53,54,55]. A marked odd-even alternating pattern was also observed: An odd number of carbon atoms tended to exhibit a lower cocrystallization efficiency [52,56,57]. However, the cause of these interesting phenomena was not unambiguously justified. As discussed earlier, our data established a clear trend that the cocrystal formability gradually diminished when the T_g_s of the structurally similar C4 diacid coformers decreased, through influencing the overall GFA of the binary systems (Figure 2). It is notable to point out that, in general, long-chain diacids (*n* = 7–10) have relatively lower melting temperatures than short-chain diacids, and accordingly, lower T_g_s, based on the empirical rule T_g_ ≈ (2/3)T_m_. Furthermore, the odd-numbered diacids have low melting temperatures and subzero T_g_s compared with their adjacent lower even-numbered diacids, owing to their inability to assume an in-plane orientation of both carboxylic groups with respect to the hydrocarbon chain [58]. Provided that T_g_ serves as a useful parameter for predicting the cocrystallization propensity, this may help shed light on why some diacids are particularly difficult to apply for forming cocrystal. Further validation of this simple prediction approach may facilitate a more rational design of novel pharmaceutical cocrystals against a large library of structurally similar coformers.

### 3.2. Effects of the Annealing Temperature and Annealing Time on the Supercooled Liquid-Mediated Re-Cocrystallization of the Amorphous VRC System: A Case Study of the VRC-TAR System

There has been a keen interest in shedding light on the phase transformations between cocrystal and amorphous systems, as well as their physical stability. Still, little is known regarding the interplay between coamorphous systems and cocrystals. Previous studies have mainly investigated the recrystallization of coamorphous systems to individual components after storage [21]. Hardly any have reported the recrystallization of coamorphous systems into the corresponding thermodynamically more stable cocrystal form [9,19]. As a prerequisite to understanding the cocrystallization from supercooled binary liquid, the effects of the (a) annealing temperature and (b) annealing time on the cocrystallization efficiency, as well as the phase purity of the obtained cocrystal, were further investigated using the VRC-TAR cocrystal as a model system.

#### 3.2.1. Annealing Temperature

**Figure 3:**
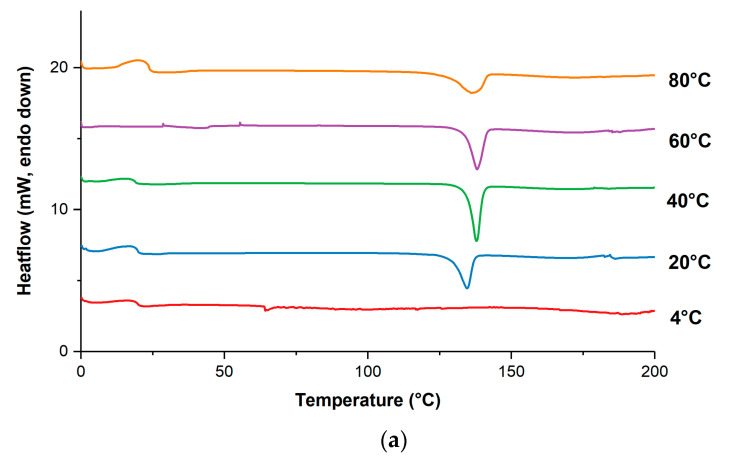

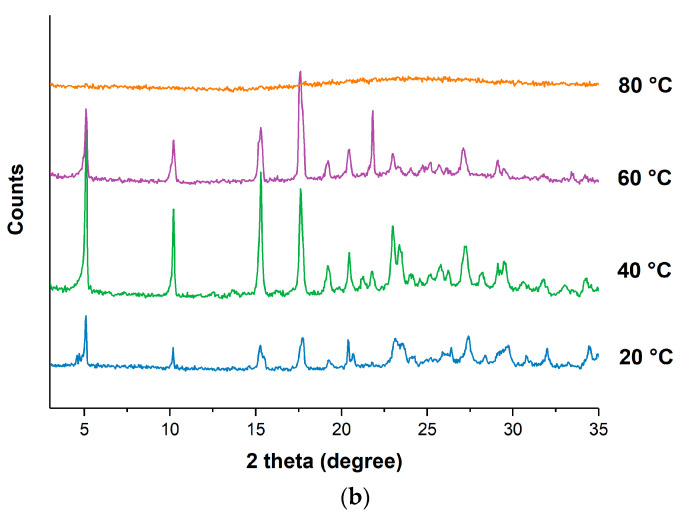
(**a**) Differential scanning calorimetry (DSC) thermograms and (**b**) powder X-ray diffraction (PXRD) patterns of the VRC-tartaric acid (TAR) system at different annealing temperatures (t_anneal_ = 3d).

The morphology of the VRC-TAR system subjected to annealing temperatures ranging from 4 to 80 °C was examined after 3-day storage (Figure S1). It is worth mentioning that cocrystallization of the amorphous VRC-TAR system appeared to happen through a supercooled liquid-mediated process, which was highly dependent on the temperature during storage. Thermal analysis and PXRD patterns of products after annealing are depicted in Figure 3a,b. Interestingly, the VRC-TAR system manifested the highest purity when the annealing temperatures were set as equal to 40 °C. The corresponding DSC profile showed a sharp melting endotherm at around 136 °C, i.e., the melting point of the phase pure 1:1 VRC-TAR cocrystal, while the PXRD data revealed the strongest intensity of the cocrystal characteristic peaks at 5.10° and 10.21° 2θ.

**Figure 4:**
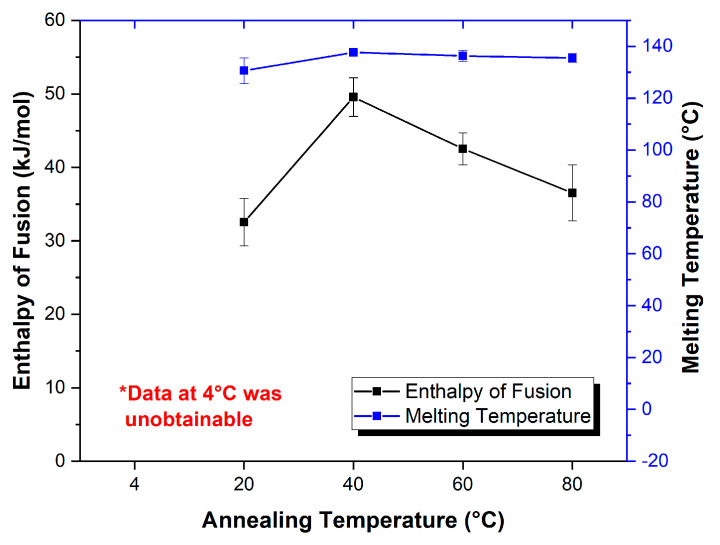
The correlation between the annealing temperature, enthalpy of fusion, and melting point of the VRC-TAR system (t_anneal_ = 3d, *n* = 3). * A storage temperature of 4 °C produced a gel-like supercooled liquid for which the melting point and enthalpy could not be identified.

It is evident that the enthalpy of fusion (kJ/mol) for the VRC-TAR cocrystal melting purity against the annealing temperature displayed a bell-shaped curve, such that temperature elevation and reduction above/below the threshold (40 °C) both inhibited the phase transformation to different extents (Table 2, Figure 4). An annealing temperature of 4 °C entirely resisted the re-cocrystallization process, with no intact melting endotherm being detected. However, when the 1:1 undercooled liquid mixture of VRC and TAR was stored at 20 °C, the DSC profiles suggested the presence of a metastable, partially crystalline state, with weaker melting endotherms located at 130.6 °C. A similar observation was also made at a high temperature, i.e., 80 °C. The result implies that the storage condition was a primary driver governing the kinetic of molecular packing, which affects the eventual occurrence of recrystallization. Fine-tuning the annealing temperature should be deemed as a particularly useful strategy for effectively obtaining hidden cocrystals, where the binary mixture of the individual coformers is associated with a relatively high GFA, such as the VRC-TAR system. For systems such as VRC-FUM with a low GFA, which could be instantly produced after rotary evaporation, the effect of the annealing temperature would be minimal (Figure S2).

It has been documented that for a single-component system, the crystallization process is intimately linked to the molecular mobility [40]. The propensity of crystallization of materials is higher above than below the T_g_, since the material is less viscous with a higher molecular mobility in a supercooled liquid state. This favors faster crystallization compared with its brittle glassy state counterpart. For a binary mixture, the relationships between the composition of the mixture and the T_g_ are commonly estimated by the Fox equation [59,60]:

(1) $$\frac{1}{{\mathrm{Tg}}_{\mathrm{mix}}}=\;\frac{w_{1}}{{\mathrm{Tg}}_{1}}+\;\frac{w_{2}}{{\mathrm{Tg}}_{2}},$$

where w_1_ and w_2_ are the weight fractions of components 1 and 2, respectively, and T_g1_ and T_g2_ are their glass transition temperatures in Kelvin, respectively. In the case of amorphous VRC-TAR, the calculated T_g_ value is 13.12 °C using the theoretical T_g_ of amorphous TAR (16 °C) and the T_g_ of amorphous VRC (11.9 °C) from the literature [27,46], which is lower than the experimental T_g_ of the VRC-TAR amorphous binary mixture (14.24 °C, Figure S3). The positive deviation from ideal behavior substantiates the formation of specific intermolecular interactions (predominately hydrogen bonding) in the amorphous VRC-TAR system upon rotary evaporation, since the Fox equation assumes that the system exhibits nearly ideal volume additivity and a negligible tendency to interact [61,62,63]. This implies a stronger binding between VRC and TAR than to themselves, leading to a lower molecular mobility. Therefore, it could be postulated that when the annealing temperature was at 4 °C, VRC-TAR would remain in a brittle glassy state with a low molecular mobility and the cocrystallization kinetic was not fast enough against amorphization. At an annealing temperature in close proximity of the T_g_ (20 °C), kinetic competition between cocrystallization and amorphization might happen. Above the T_g_ (40 and 60 °C), the amorphized content of VRC-TAR became lower and more rapid crystallization towards the cocrystal form was facilitated by the higher molecular mobility in supercooled liquid. However, one should note that a further increase in the annealing temperature over the optimum might generate spatial disorder and result in a structure consisting of randomly oriented coformers due to the very high molecular mobility.

#### 3.2.2. Annealing Time

**Figure 5:**
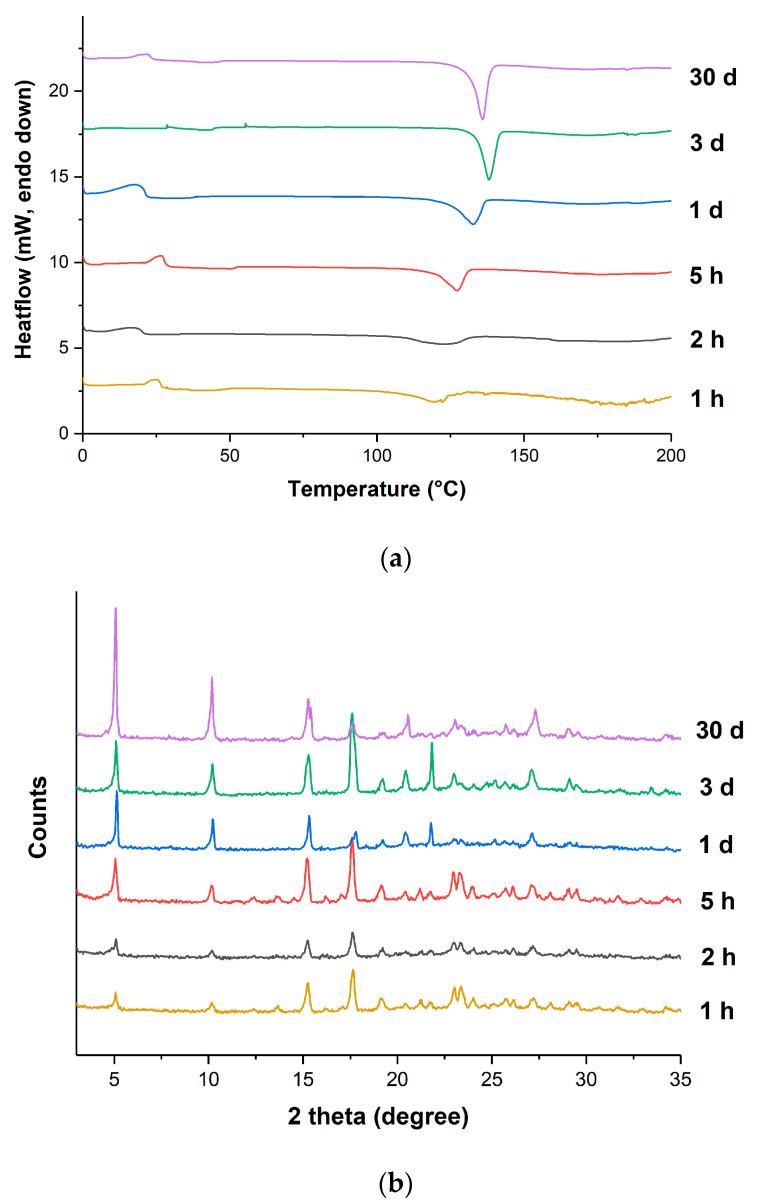
(**a**) DSC thermograms and (**b**) PXRD patterns of the VRC-TAR system at different annealing time points (T_anneal_ = 60 °C).

**Figure 6:**
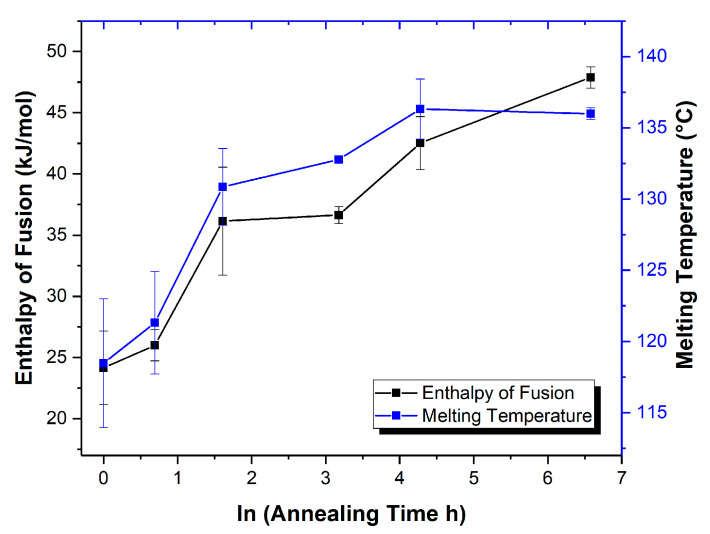
Elevation of melting enthalpy throughout annealing of the VRC-TAR system (T_anneal_ = 60 °C, *n* = 3).

The short-term physical stability of the VRC-TAR cocrystal system was subsequently tested with a constant annealing temperature of 60 °C for the periods of 1h, 2 h, 5 h, 1 d, 3 d, and 30 d. It is noticeable that the DSC peak position of the product shifted to a higher temperature until reaching a threshold of ~136 °C on the 3rd day of aging, which is the melting point of pure VRC-TAR cocrystal (Figure 5a). In general, the enthalpy of fusion (kJ/mol) corresponding to VRC-TAR cocrystal melting increased with an increase of the annealing time, indicating improvement of the cocrystal phase purity via the formation of stronger intermolecular hydrogen bonding between the API and the coformer (Figure 6 and Figure S4). The PXRD data also showed a gradual enhancement in the integral intensity of the cocrystal characteristic peaks at 5.10° and 10.21° 2θ (Figure 5b). These suggest the existence of an intermediate coamorphization stage preceding the transformation towards the thermodynamically more stable cocrystalline phase. It is plausible that cocrystallization via rapid solvent removal, i.e., rotary evaporation, is generally a multi-step process. The kinetic entrapment of VRC cocrystal molecules initially resulted in an unwanted transient amorphous state with a high instability, as predicted by Ostwald’s rule of successive stages [7]. This short-lived metastable species converted into cocrystal at a specific timescale under appropriate processing conditions, which was dependent on the annealing temperature, as well as the GFA of the API and coformer.

The kinetics of the amorphous-to-cocrystal phase transformation of VRC-TAR at 60 °C was also investigated by analyzing the time-dependent change in the relative crystallinity (%RC) of the product obtained, which was calculated as the peak area of the 5.10° and 10.21° 2θ characteristic peaks at different annealing time points (PA[t]) divided by those at 30 d-annealing (i.e., the highest attainable crystallinity at 60 °C) (Table 3). High-angle Bragg peaks were excluded for comparison since it is generally agreed that high-angle XRD data generate poorer counting statistics, which can be attributed to the combined effects of a decrease in the scattering coefficient with increasing sin θ/λ, the Lorentz–polarization factor, and thermal vibrations [64]. The isothermal nucleation and growth process of VRC-TAR can be fitted into the Kolmogorov–Johnson–Mehl–Avrami (KJMA) equation [65,66,67]. The KJMA equation is a classical kinetics theory applied in a variety of metals and pharmaceutical compounds for describing the phase transformation between crystalline and amorphous phases:

(2) $$y=1-\exp\left(-kt^{n}\right),$$

where y is the transformed phase fraction; *k* is the overall rate constant depending on the temperature; t is the time; and *n* is the Avrami exponent, which provides a qualitative indication of the mechanisms of the nucleation processes and crystal growth. The transformed phase fraction (y) is represented by the calculated %RC of the cocrystal. Since the equation is usually rewritten as

(3) ln(−ln[1−y])=ln(k)+nln(t),

the experimental data are then plotted as ln(−ln[1−y]) against ln(t) for tracing the transformation’s underlying mechanisms.

**Figure 7:**
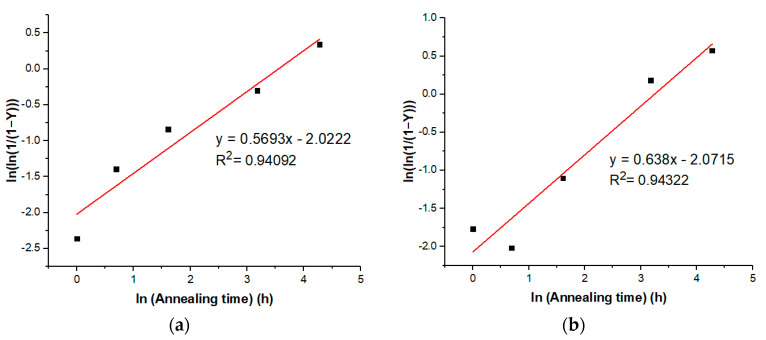
Isothermal phase transformation plots for the VRC-TAR system (T_anneal_ = 60 °C), as described using the Avrami equation based on the (**a**) %RC at 5.10° 2θ and (**b**) %RC at 10.21° 2θ.

**Figure 8:**
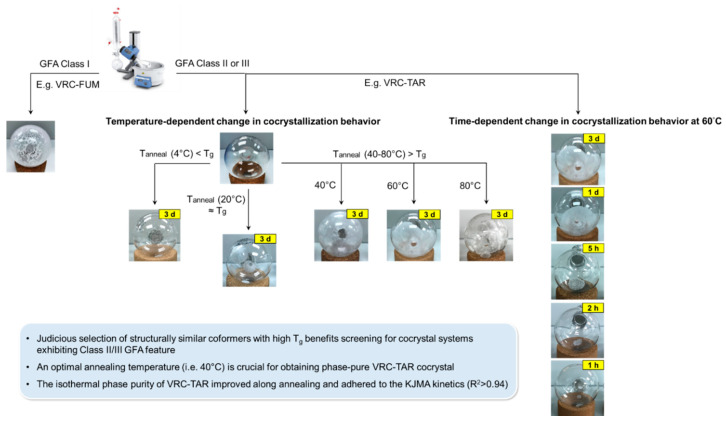
Schematic of VRC-FUM and VRC-TAR cocrystals formation via rotary evaporation.

As shown in Figure 7a,b, the cocrystallization expresses an Avrami-like kinetics, yielding a straight line with slope *n* and intercept ln(k) (R^2^ > 0.94). Therefore, the Avrami exponent and the reaction rate could be deduced as 0.604 and 0.129 (average values obtained from 5.1° and 10.21° 2θ), respectively. In fact, it is not uncommon to obtain an effective Avrami exponent with values lower than 1 in the literature [68]. For instance, Lin et al. reported a relatively low Avrami exponent of 0.37 for nanocrystalline copper prepared by dynamic plastic deformation owing to the effect of heterogeneity of the deformation microstructure on the recrystallization kinetics [69]. In the case of VRC-TAR, this could also be reasonably attributed to the presence of a non-zero transformed fraction at the starting point of annealing, since there might exist a trace amount of instantaneous microcrystalline growth at the boundaries of the original phase upon the completion of rotary evaporation [68]. Despite the limitations of applying the KJMA model to the supercooled liquid-mediated re-cocrystallization, the well-established framework renders it appealing for extracting useful kinetic parameters for preliminary analysis and a system-to-system comparison. An overview of the interplay between the GFA, annealing conditions, and cocrystallization behavior of the VRC systems is summarized in Figure 8.

### 3.3. Physical Characterization of the VRC-TAR Cocrystal

**Figure 9:**
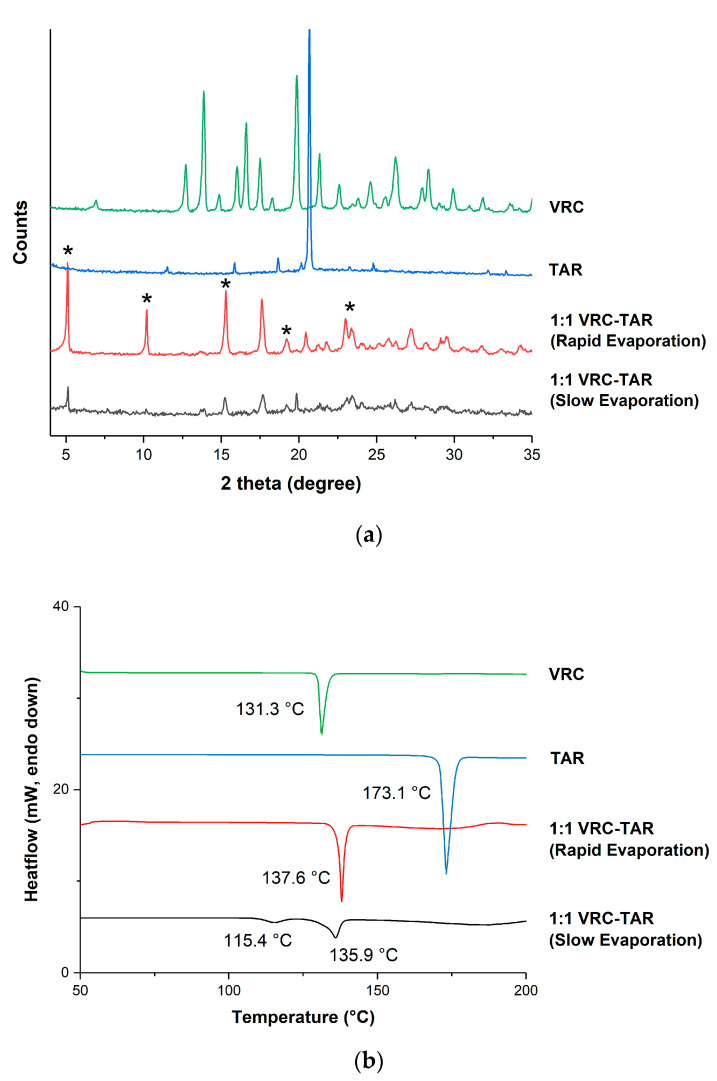

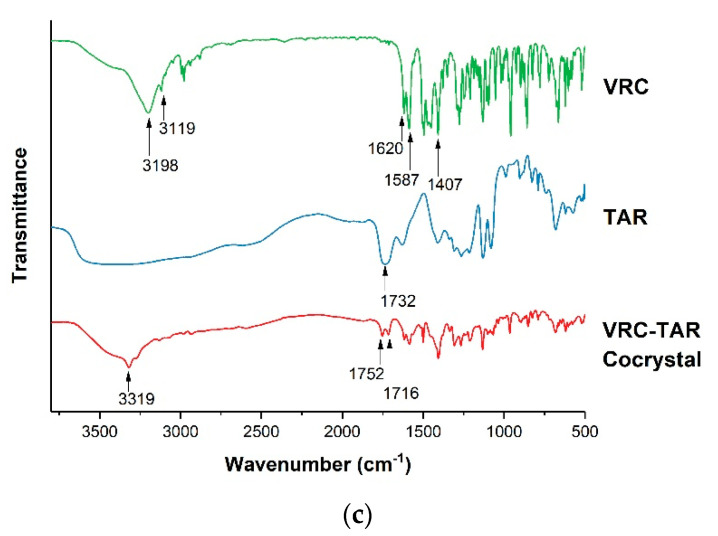
Solid-state characterization of the VRC-TAR cocrystal system: (**a**) PXRD patterns, new peaks are marked with *; (**b**) DSC thermograms; and (**c**) Fourier-transform infrared spectroscopy (FTIR) patterns.

A better understanding of the physicochemical properties of the newly synthesized VRC-TAR cocrystal is desirable. The formation of this new phase was confirmed by PXRD, DSC, and FTIR, respectively. The PXRD patterns of the VRC-TAR sample displayed a number of distinct diffraction peaks (2θ = 5.10°, 10.21°, 15.29°, 19.19°, 22.98°, and 23.38°), while characteristic peaks (VRC: 12.72°, 13.88°, 16.02°, 16.61°, and 19.85° 2θ; TAR: 11.54°, 15.86°, 18.67°, and 20.68° 2θ) corresponding to VRC and TAR were absent (Figure 9a). No solid-state polymorphic change of VRC was observed by rapid evaporation on the basis of the XRD diffractograms (Figure S5). Figure S6 shows the PXRD patterns of the VRC-FUM, VRC-MAE, and VRC-MAL systems. The PXRD pattern of the VRC-FUM cocrystal reproduced by rotary evaporation is consistent with that reported in the literature [30], while those of the other two systems were simply superposition of the cocrystal formers.

Regarding thermal analysis, the DSC thermograms of VRC, C4 diacid coformers, and the corresponding binary products obtained by rotary evaporation are illustrated in Figure 9b and Figure S7. The data of VRC-TAR confirmed the presence of a homogeneous solid phase with a high phase purity, showing a sharp single endotherm at 137.6 °C. This could be assigned to the melting point of the cocrystal, which lies between those of VRC (131.3 °C) and TAR (173.1°C), and thus excluding the possibility of a eutectic formation. The cocrystal only existed at a 1:1 molar ratio. The binary mixtures of TAR with varying VRC mole fractions in the range of ~0.2 to 0.4 were retained as a gel-like mass and thus posed a challenge in constructing a temperature–composition phase diagram through thermal analysis. The crystal lattice strengthening effect upon cocrystallization was revealed by the elevated enthalpy of fusion (ΔH_f_) of the VRC-TAR cocrystal (49.56 kJ/mol) compared with its starting materials (VRC: 32.4 kJ/mol, TAR: 36.0 kJ/mol). In contrast, slow solvent evaporation in ethanol resulted in incomplete conversion to the cocrystal (Figure 9a,b). This could be ascribed to the incongruent solubility of VRC (63.0 ± 0.9 mg/mL) and TAR (272.5 ± 6.4 mg/mL) in the crystallization solvent, reflecting the propensity of the less soluble former, i.e., VRC, to preferentially crystallize out from solution before it reaches the labile zone for spontaneous cocrystallization [1].

The overlaid FTIR spectra for the VRC-TAR cocrystal system are presented in Figure 9c. In correspondence with the literature [70,71], VRC showed a broad peak at 3198 and 3119 cm^–1^ attributed to the O–H stretching and aromatic rings (amine N–H stretch). Sharp peaks at 1620 and 1587 cm^–1^ are indicative of C=N stretching and aromatic C=C stretching, respectively, while the presence of the C–F bond is assigned to the peak at 1407–1095 cm^–1^. The spectra of TAR displayed characteristic C=O stretching at 1732 cm^–1^ and an absorption band in the region of ~3500–2700 cm^–1^ as a result of a broad O–H band superimposed on the C–H stretching. Spectral peak shifts were observed for various functional groups in VRC-TAR cocrystal, suggesting an alteration of the chemical environment of the solid state. Compared with pure VRC, the phenolic O−H stretching frequency of VRC-TAR dramatically upshifted to 3319 cm^–1^, which implies the formation of an O–H⋯N heterosynthon between the carboxyl group of TAR with the torsionally flexible triazole or pyrimidine N atoms in VRC [30]. The C=N and C=C stretches in cocrystal were unaffected.

### 3.4. Dissolution Performance

VRC is currently available on the market as an intravenous infusion solution and two oral formulations, film-coated tablets, and suspensions [72]. Despite its broad-spectrum of activity compared with its structural congener fluconazole, VRC exhibits a lower aqueous solubility (<0.1 mg/mL) and non-linear pharmacokinetics associated with a narrow therapeutic range [25,73,74]. Approximately 100-fold inter-patient variability in plasma drug levels was reported, which implies that standard oral VRC doses might be inadequate for achieving an effective concentration in some cases, especially in critically ill patients [73,75]. Given that VRC is a poorly water-soluble weak base, one of the factors associated with the erratic absorption could be the physiological change in fluid pH, composition, and volume after gastrointestinal transfer [76,77]. The highly pH-dependent solubility renders VRC susceptible to intestinal precipitation as the pH elevates upon entry of the small intestine, where the absorption takes place. As the cocrystal solubility has been shown to be directly proportional to the solubility of constituent reactants (i.e., the drug and coformer) [55], the cocrystallization of a poorly water-soluble API with a water-soluble coformer is expected to offer a dissolution advantage in formulation.

**Figure 10:**
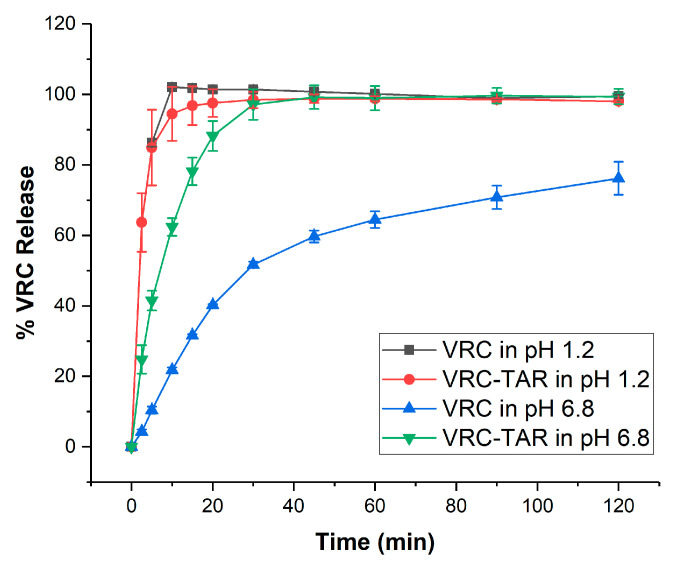
Dissolution profiles of the VRC-TAR cocrystal system at pH 1.2 and pH 6.8.

To this end, we sought to compare the dissolution profiles of the VRC-TAR cocrystal against raw VRC using compendial buffer media at pH 1.2 and pH 6.8. As shown in Figure 10, the VRC and VRC-TAR cocrystal both underwent rapid dissolution at pH 1.2. Nonetheless, the release of VRC was substantially reduced at pH 6.8. At 45 min, i.e., the end point of the VRC dissolution test recommended by the US FDA, all VRC dissolved at pH 1.2, whereas only 59.7% was released at pH 6.8, and therefore, it is estimated that a significant portion of VRC would precipitate in the intestine after gastric emptying. On the other hand, the VRC-TAR cocrystal conferred superior dissolution enhancement over VRC at pH 6.8, as an initial burst release of 41.5% was observed at 5 min, and it reached a plateau at a 95% VRC fraction of release after 30 min. The release profile followed first-order kinetics in the beginning 15 min (R^2^ = 0.95). The release of VRC was maintained for up to 2 h, without any precipitation. Through minimizing the VRC concentration difference between the dissolution behaviors of VRC-TAR cocrystal at pH 1.2 (red line) and pH 6.8 (green line), the driving force for precipitation could be lessened in the gastrointestinal tract.

Since the VRC-TAR cocrystal and raw VRC were sifted within the same size range to negate the effects of the particle size and morphology upon dissolution (Figure S8), the intrinsic dissolution rate (IDR) can be roughly estimated based on the slope of the initial linear region of the cumulative dissolution curve, i.e., (dm/dt)max, using the equation IDR = (dm/dt)max/A, where A is the specific surface area of the dissolution sample. The IDR ratio of VCR-TAR to VRC is 5.76, resulting in a ~6-fold enhancement in the rate of dissolution of the cocrystal in comparison to raw VRC in the intestinal environment. Hence, the cocrystallization of VRC with TAR substantially improved the dissolution performance of VRC at pH 6.8 due to the higher aqueous solubility of TAR, i.e., 206 mg/mL at 20 °C [78].

## 4. Conclusions

Amorphous solids, incorporating an active pharmaceutical ingredient and a coformer, are often encountered during cocrystal screening via rapid solvent removal. This study delineated the effects of the coformer’s GFA and annealing conditions on the re-cocrystallization behavior of an amorphous system. Careful manipulation of the annealing temperature and time allowed the effective production of a phase pure 1:1 VRC-TAR cocrystal from its amorphous counterpart via a supercooled-mediated re-cocrystallization process. The cocrystal exhibited a 6-fold increase of the dissolution rate, which may mitigate the problematic issue of the erratic bioavailability of VRC by preventing its precipitation at an intestinal pH. Further investigation is warranted to determine whether the strategy could revive the previously failed attempts at a target cocrystal, and therefore expand the pharmaceutical solid form diversity.

## Figures and Tables

**Table 1:** Thermal properties of VRC and C4 diacid coformers.

Chemical Name	M.W. (g/mol)	M.P. (°C)	ΔH_f_ (kJ/mol)	T_g_ (°C)
VRC	349.3	131.4	32.4	11.9 ^1^
FUM	116.1	294.4	56.0	105.2 ^2^
TAR	150.1	173.1	36.0	16 ^3^
MAE	116.1	143.4	30.9	4.6 ^2^
MAL	134.1	101.1	22.1	−20 ^3^

^1^ Ref. [27], ^2^ calculated using T_g_ = (2/3)T_m_ rule, and ^3^ ref. [46].

**Table 2:** Temperature-dependent change in the thermal properties of the VRC-TAR system after 3-day annealing.

Annealing Temp. (°C)	M.P. (°C)	ΔH_f_ (kJ/mol)
4	− ^1^	− ^1^
20	130.6 ± 5.0	32.5 ± 3.2
40	137.6 ± 0.5	49.6 ± 2.6
60	136.3 ± 2.1	42.5 ± 2.2
80	135.5 ± 1.7	36.5 ± 3.8

^1^ An annealing temperature of 4 °C produced a gel-like supercooled liquid for which the melting point and enthalpy could not be identified.

**Table 3:** Time-dependent change in the relative crystallinity (%RC) of the VRC-TAR cocrystal at an annealing temperature of 60 °C.

Annealing Time	PA[t] at 5.10° 2θ	%RC at 5.10° 2θ ^1^	PA[t] at 10.21° 2θ	%RC at 10.21° 2θ ^1^
1 h	3.1	9.0	3.3	15.7
2 h	7.5	22.0	2.6	12.4
5 h	12.0	35.0	5.9	28.3
1 d	18.0	52.4	14.7	69.9
3 d	25.9	75.4	17.5	83.1
30 d	34.4	100	21.0	100

^1^ RC of the VRC-TAR cocrystal = PA[t]/PA [30 d]; t = annealing time.

## Supplementary Materials

The following are available online at https://www.mdpi.com/1999-4923/12/12/1209/s1: Figure S1: The morphology of the VRC-TAR system presented in a round bottom flask at different annealing temperatures (t_anneal_ = 3d); Figure S2: The melting temperatures and morphologies of the VRC-FUM system at different annealing temperatures (t_anneal_ = 3d); Figure S3: T_g_ determination for the VRC-TAR system through a DSC heat-cool-heat cycle. Green line: Heating (10 °C/min); blue line: Quench cooling (50 °C/min); black line: Re-heating (10 °C/min); Figure S4: The morphology of the VRC-TAR system presented in a round bottom flask at different annealing time points (T_anneal_ = 60 °C); Figure S5: PXRD patterns of VRC pre- and post-rotary evaporation; Figure S6: PXRD patterns of VRC-FUM, VRC-MAE, and VRC-MAL systems produced by rotary evaporation; Figure S7: DSC profiles of VRC-FUM, VRC-MAE, and VRC-MAL systems produced by rotary evaporation (* regarded as the degradation peak of VRC-MAL); and Figure S8: Optical micrographs of the (a) sifted VRC and (b) VRC-TAR cocrystal at a magnification of 40x.
