# Supplementary material for: Effects of the Glass-Forming Ability and Annealing Conditions on Cocrystallization Behaviors via Rapid Solvent Removal: A Case Study of Voriconazole
Source: Pharmaceutics. 2020 Dec 14;12(12):1209. doi: 10.3390/pharmaceutics12121209 (PMC7764899; doi:10.3390/pharmaceutics12121209)
Supplement: Supplementary file 1 [file pharmaceutics-12-01209-s001.pdf]

pharmaceutics

MDPI

Supplementary Materials: Effects of the Glass-Forming Ability and Annealing Conditions on Cocrystallization Behaviors via Rapid Solvent Removal: A Case Study of Voriconazole

Si Nga Wong, Susan Wing Sze Chan, Xuexin Peng, Bianfei Xuan, Hok Wai Lee, Henry H.Y. Tong and Shing Fung Chow

Annealing Temp.	Physical Appearance of Products
4°C	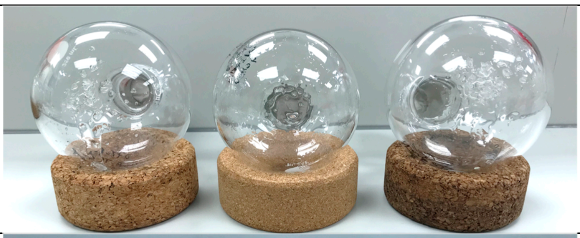
20°C	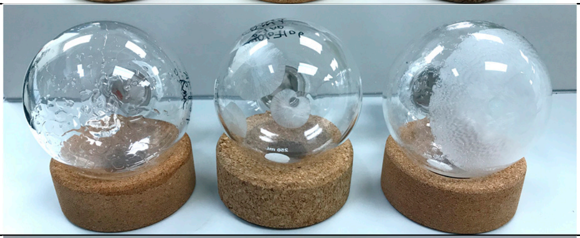
40°C	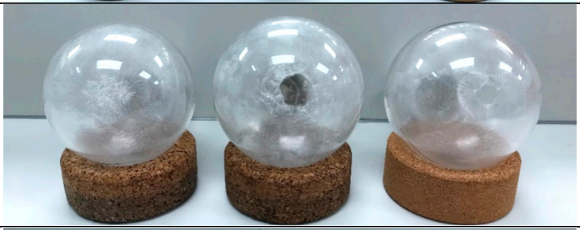
60°C	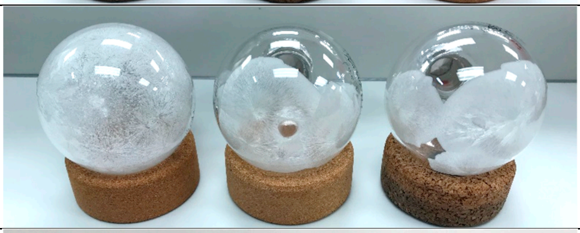
80°C	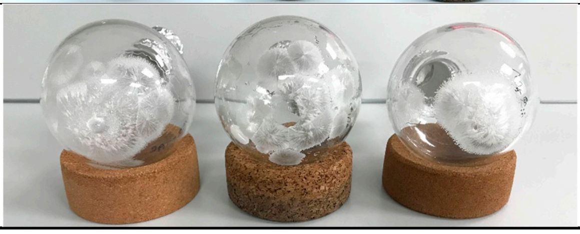

Figure S1. The morphology of VRC-TAR system presented in round bottom flask at different annealing temperatures (t_{anneal}= 3d).

Annealing Temperature (°C)	20°C	60°C	80°C
Melting Temperature (°C)	136.8	135.1	137.4
Physical Morphology	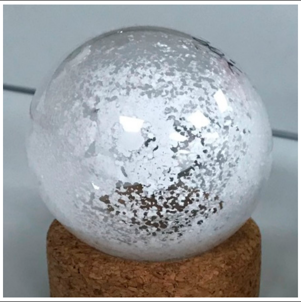	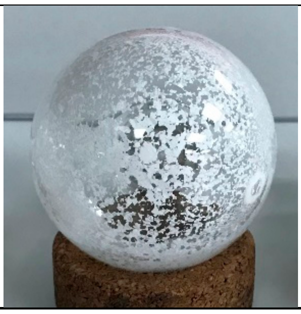	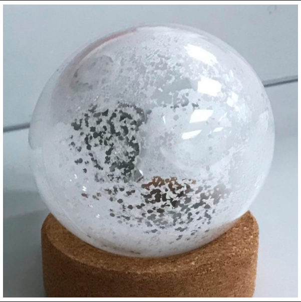

Figure S2. The melting temperatures and morphologies of VRC-FUM system at different annealing temperatures (t_{anneal}= 3d).

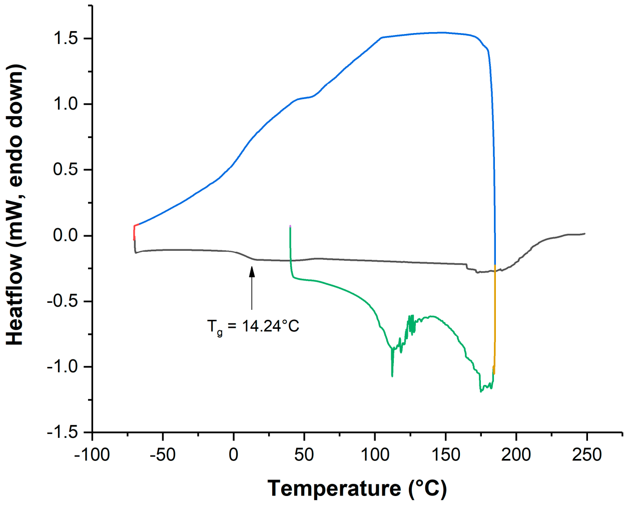

Figure S3. T_g determination for VRC-TAR system through DSC heat-cool-heat cycle. Green line: Heating (10 °C/min); Blue line: Quench cooling (50 °C/min); Black line: Re-heating (10 °C/min).

Annealing Time	Physical Appearance of Products
1 h	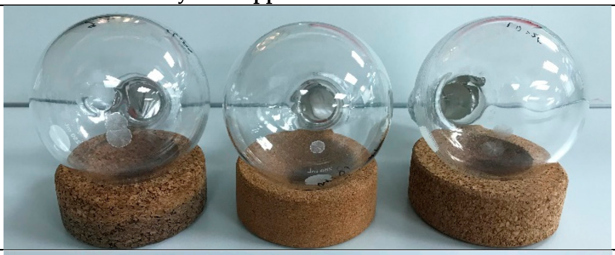
2 h	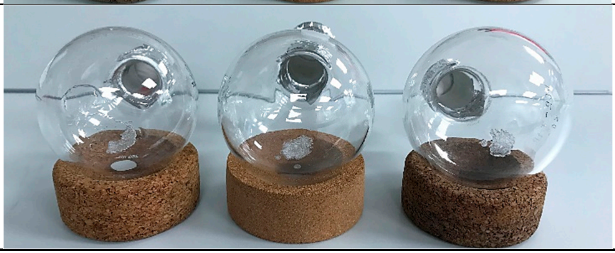

**5 h**	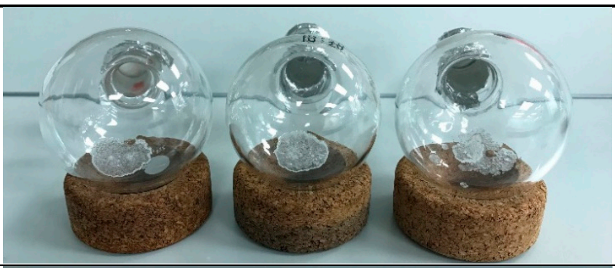
**1 d**	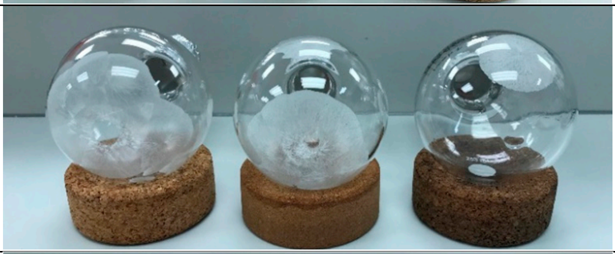
**3 d**	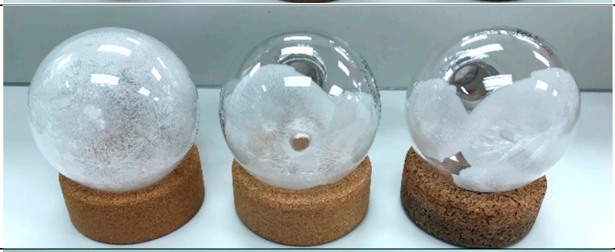
**30 d**	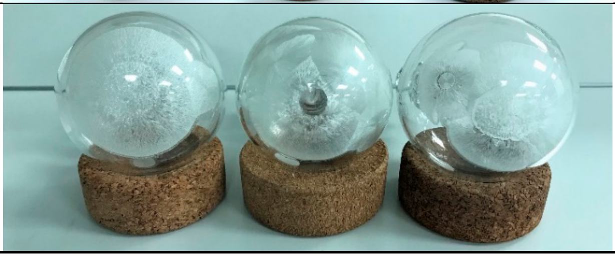

Figure S4. The morphology of VRC-TAR system presented in round bottom flask at different annealing time points (T_{anneal}= 60°C).

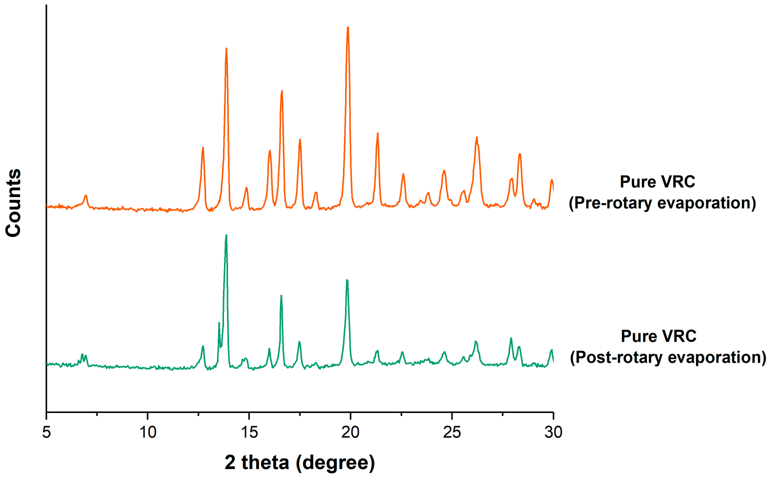

Figure S5. PXRD patterns of VRC pre- and post-rotary evaporation.

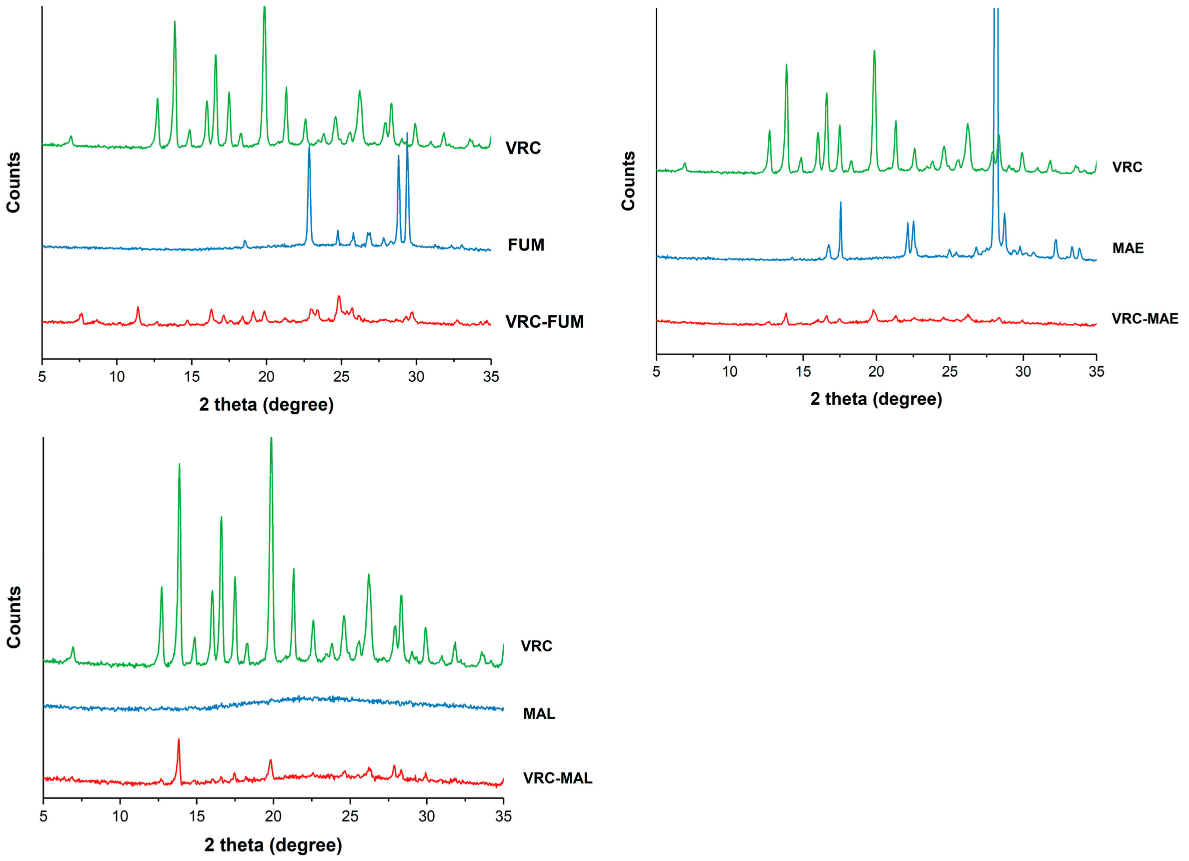

Figure S6. PXRD patterns of VRC-FUM, VRC-MAE, and VRC-MAL systems produced by rotary evaporation.

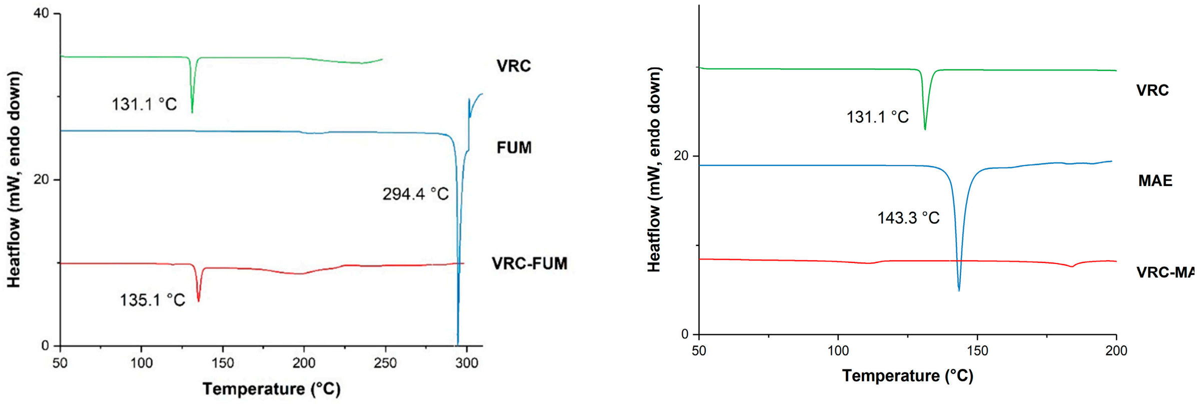

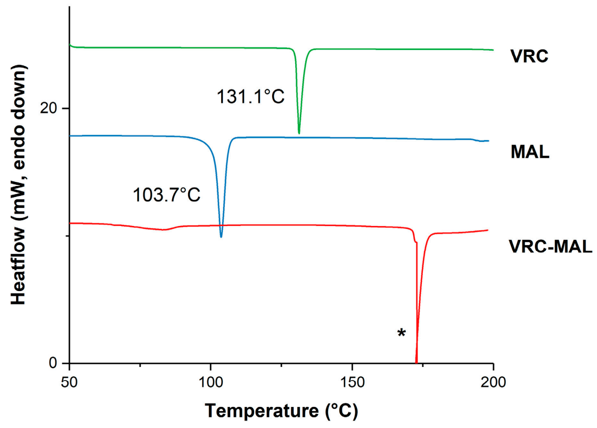

Figure S7. DSC profiles of VRC-FUM, VRC-MAE, VRC-MAL systems produced by rotary evaporation (* regarded as the degradation peak of VRC-MAL).

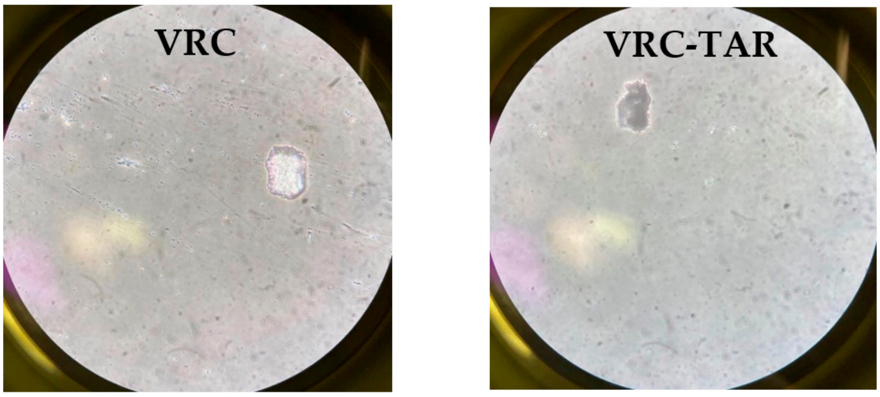

(**a**) (**b**)

Figure S8. Optical micrographs of the (a) sifted VRC and (b) VRC-TAR cocrystal at a magnification of 40x.
